# Malignant canine mammary epithelial cells shed exosomes containing differentially expressed microRNA that regulate oncogenic networks

**DOI:** 10.1186/s12885-018-4750-6

**Published:** 2018-08-20

**Authors:** Eric J. Fish, Kristopher J. Irizarry, Patricia DeInnocentes, Connor J. Ellis, Nripesh Prasad, Anthony G. Moss, R. Curt Bird

**Affiliations:** 1 Department of Pathobiology, College of Veterinary Medicine, Auburn University, 166 Greene Hall, Auburn, AL 36849 USA; 2 College of Veterinary Medicine, Western University of Health Sciences, Pomona, CA 91766 USA; 3 Genomic Services Laboratory, Hudson Alpha Institute for Biotechnology, Huntsville, AL 35806 USA; 4 Department of Biology, College of Science and Mathematics, Auburn University, Auburn, AL 36849 USA

**Keywords:** Canine, Mammary cancer, microRNA, miR-18a, Estrogen receptor, Exosome, Exosomal, Deep-sequencing, RNAseq, Bioinformatics, Translational

## Abstract

**Background:**

Breast (mammary) cancers in human (BC) and canine (CMT) patients share clinical, pathological, and molecular similarities that suggest dogs may be a useful translational model. Many cancers, including BC, shed exosomes that contain microRNAs (miRs) into the microenvironment and circulation, and these may represent biomarkers of metastasis and tumor phenotype.

**Methods:**

Three normal canine mammary epithelial cell (CMEC) cultures and 5 CMT cell lines were grown in serum-free media. Exosomes were isolated from culture media by ultracentrifugation then profiled by transmission electron microscopy, dynamic light scattering, and Western blot. Exosomal small RNA was deep-sequenced on an Illumina HiSeq2500 sequencer and validated by qRT-PCR. In silico bioinformatic analysis was carried out to determine microRNA gene and pathway targets.

**Results:**

CMEC and CMT cell lines shed round, “cup-shaped” exosomes approximately 150–200 nm, and were immunopositive for exosomal marker CD9. Deep-sequencing averaged ~ 15 million reads/sample. Three hundred thirty-eight unique miRs were detected, with 145 having > ±1.5-fold difference between one or more CMT and CMEC samples. Gene ontology analysis revealed that the upregulated miRs in this exosomal population regulate a number of relevant oncogenic networks. Several miRNAs including miR-18a, miR-19a and miR-181a were predicted in silico to target the canine estrogen receptor (ESR1α).

**Conclusions:**

CMEC and CMT cells shed exosomes in vitro that contain differentially expressed miRs. CMT exosomal RNA expresses a limited number of miRs that are up-regulated relative to CMEC, and these are predicted to target biologically relevant hormone receptors and oncogenic pathways. These results may inform future studies of circulating exosomes and the utility of miRs as biomarkers of breast cancer in women and dogs.

**Electronic supplementary material:**

The online version of this article (10.1186/s12885-018-4750-6) contains supplementary material, which is available to authorized users.

## Background

Canine mammary tumors (CMT) represent the most frequent tumor in hormonally intact (non-ovariohysterectomizd) female dogs, and CMT have similar incidence and comparable distribution of malignant potential to breast cancer (BC) in women [1–3]. Some forms of CMT may represent a useful translational model for human BC as it shares risk factors such as age, hormone exposure, and obesity, although there are some differences in subtypes that limit direct comparison [4–6]. Furthermore, CMT and BC share similar genetic alterations, including downregulation of tumor suppressors p16/INK4A, PTEN, BRCA1, and p53, as well as upregulation of oncogenes KRAS, PI3K/AKT, and MAPK [7–9]. Recently, CMT has been shown to be classifiable into human molecular subtypes luminal A, luminal B, HER-2, and triple-negative/basal-like according to estrogen receptor alpha (ESR1α), progesterone receptor, and HER-2/ErbB-2 expression by immunohistochemistry in patient tumor tissue and by qRT-PCR in a cohort of well-characterized cell lines [10, 11]. In addition, CMT, like human BC, shows a negative correlation between estrogen hormone receptor ESR1 expression and increasing tumor grade [10, 12].

Currently, as in women with BC, definitive classification of benign versus malignant CMT, as well as tumor grading, requires histopathology. This is problematic because collecting those samples requires invasive surgery. Current less invasive alternatives such as fine-needle aspirate cytology vary from 67.5–81% accuracy [13, 14]. Another significant prognostic factor for CMT is advanced stage, with shortened survival times for dogs with large tumors (> 3 cm) and/or metastasis, highlighting the importance of early detection [15, 16]. An accurate, minimally invasive, biomarker for CMT diagnosis and malignant potential could improve outcomes through intervention at a lower stage of disease.

One such potential class of biomarker is microRNAs (miRNAs, miRs), a type of small (18–22 nucleotides), non-coding RNA that are highly conserved across species and play crucial roles in the negative, post-transcriptional regulation of gene expression in both health and disease [17]. Each miRNA recognizes numerous gene targets through hybridization with a complementary “seed sequence” in the 3′ untranslated region (UTR) of mRNA resulting in either degradation of the transcript or inhibition of ribosomal translation [18]. Dysregulation of miRNAs is particularly prevalent in cancer, where genetic instability of tumors leads to altered miRNA expression profiles that promote oncogenesis [19]. Numerous studies demonstrate miRNA are differentially expressed in women with BC in tissue, exosomes, and serum/plasma, [20–23].

Multiple miRNAs are already known to be altered in CMT, though the data are complex and sometimes conflicting, particularly depending on the RNA source (cells, exosomes, tumor tissues, serum/plasma, etc), microRNA profiling technique(s), and normalizing strategies. One early study of miRNA expression between CMT and normal mammary gland tissue using a small set of individual qRT-PCR assays found miR-29b and miR-21 were significantly upregulated in neoplastic versus normal tissue, while miR-181b and let-7f were specifically upregulated in tubulopapillary carcinoma [24]. In another, miR-141 specifically was demonstrated to be upregulated in several well-characterized CMT cell-lines and experimentally validated to down-regulate tumor suppressor p16/INK4a [9]. This same study also identified a number of other differentially expressed miRs by qRT-PCR microarray in CMT that were also altered in human BC, including miR-21, miR-155, miR-9, miR-34a, miR-143/145, and miR-31 [9]. A separate research group established a CMT line (labeled “SNP”) from a primary patient mammary tumor and compared its miRNA expression to normal mammary tissue through miRNA hybridization arrays and found the microRNAs with the greatest increase and decrease were miR-143 and miR-138a, respectively [25]. A separate study of various primary and metastatic canine mammary tumor tissues using qRT-PCR found up-regulated miR-210 in neoplastic versus normal tissue, higher miR-21 in malignant mammary carcinomas (but not benign tumors), and that the metastatic tumors had altered miR-29b, miR-101, mir-125a, miR-143 [26]. Another study evaluated primary versus metastatic mammary carcinomas using RNA hybridization arrays and qRT-PCR, and found a distinct signature of microRNA expression in metastatic canine mammary carcinoma, although the expression of these candidate metastasis markers were not statistically different in peripheral blood [27]. Finally, a recent study evaluating circulating microRNA in blood by qRT-PCR for multiple different types of cancer found miR-214 and miR-126 were significantly up-regulated in serum from dogs with mammary carcinoma (along with numerous other tumor types) [28]. Of note, most of these studies performed no or limited in silico bioinformatics analysis for these miRNA, and only the study of miR-141 and p16/INK4a experimentally validated the annotated targets [9].

miRNAs make particularly good biomarkers because they can be secreted in biofluids such as serum, urine and breast milk, and protected from endogenous RNases by packaging in exosomes and/or binding to proteins such as Argonaute [17, 18]. Exosomes are 30–200 nm in diameter round vesicles with a lipid membrane, and are secreted by cellular organelles called multivesicular bodies. There is some evidence to indicate that exosomes are actively secreted by tumor cells to facilitate cell-to-cell communication to distant cells and tissues [29]. These tumor exosomes and their cargo of miRs, mRNA, and proteins may also modulate the behavior of local stromal and immune cells [30]. One such study provided data that tumor exosomes derived from human patients with lung and pancreatic carcinomas were able to induce myotube apoptosis through miR-21 and TLR7 signaling, recapitulating the cancer cachexia phenotype in an in vitro model [31].

The aims of the current study were to isolate and characterize exosomes shed by normal canine mammary epithelial cells (CMEC) and CMT cells in vitro, analyze the miRNA profile of these exosomes, and to perform in silico bioinformatic annotation of this miRNA signature. We hypothesized that both CMEC and CMT cells grown in serum-free media would shed exosome-like microvesicles containing abundant miRNAs, and that the miRNA signature of the CMT extracellular vesicles would be enriched in a subpopulation of miRs predicted to regulate important molecular targets in CMT.

## Methods

### Cell culture

The following cell lines were used: Three normal canine mammary epithelial cell cultures independently derived from separate canine patients without mammary pathology (CMEC1, CMEC2, CMEC3), and five stable and highly transformed cell lines derived from canine patients with histopathology-confirmed mammary carcinoma including CMT12 (formerly CMT2), CMT27 (formerly CMT4) and CMT28 (formerly CMT5) as well as 2 more recently derived lines including CMT47 (derived from a mammary adenocarcinoma from a pure-bred Miniature Schnauzer) and CMT119 (derived from a mammary carcinoma from a Golden Retriever) [9, 11]. The CMT cells used are the product of our laboratory group in collaboration with Dr. Lauren Wolfe (retired). They were all derived/rescued from surplus biopsy specimens recovered following standard of care surgery of canine mammary cancer patients. Each biopsy specimen to be cultured was divided in two at the time of collection: one for epithelial cells to be sorted by flow cytometry and grown in culture, and one placed in formalin to be processed for routine histopathology and reviewed by a board-certified pathologist to identify the cell type and confirm malignancy. All CMT cell lines in this study were confirmed to be derived from mammary carcinoma/adenocarcinoma tumors on histopathology, but tissues were not further classified into mammary tumor histologic subtypes (i.e. simple, complex, micropapillary, etc). All owners of such animals sign a general informed consent that notes that biopsy specimens recovered in this manner may be used for research. No IACUC approval is required for such specimens. All CMEC samples were recovered from normal geriatric female dogs in the breeding colony housed and managed under IACUC PRN 2015–2688. All such specimens were recovered post-mortem following euthanasia performed in the normal management of the colony. In no case were any of these animals euthanized for the current study. All cell lines are routinely analyzed using canine-specific RT-PCR assays for Canine Mammaglobin-A (unpublished data) to ensure the species source.

CMT and CMEC cells were grown in 75 cm^2^ flasks in synthetic Xerum-free® media + DMEM media supplemented with 2X penicillin/streptomycin antibiotics at 37 °C until 70–80% confluence. Media from the first 24 h of culture was discarded and conditioned media from the second 48 h was harvested on day 3 of growth prior to trypsinization and subculture.

### Exosome isolation

Exosomes and exosomal proteins were isolated by progressive centrifugation and ultracentrifugation. Briefly, 5–10 mL conditioned cell culture media were progressively centrifuged at 4 °C at 300×g for 10 min, 2000 x g for 10 min, and 10,000 x g for 30 min, each time discarding the pellet and retaining the supernatant, to remove cells and debris. The processed supernatants were then centrifuged at 100,000 x g at 4 °C for 70 min, and the resulting supernatant was subjected to another cycle of centrifugation at 100,000 x g at 4 °C for 70 min. The final pellet was resuspended in 50 uL PBS [32].

### Dynamic light scattering

The size distribution of vesicles in the cell-free conditioned media diluted 1:5 to 1:20 (depending on particle count rate) in 1× DEPC-treated PBS was measured by intensity-weighted dynamic light scattering using a Malvern ZetaSizer ZS90 (Malvern instruments, Ltd., Worcestershire, UK) according to the manufacturer’s instructions.

### Transmission electron microscopy

Cell-free conditioned media from both CMEC and CMT cells, prepared by progressive centrifugation and ultracentrifugation as previously described, was loaded onto copper-formvar grids treated with 1% Alcian blue (to increase hydrophobicity) and negatively stained with 1% uranyl acetate [32]. Grids were loaded into a Zeiss EM10 transmission electron microscope (Carl Zeiss Microscopy, LLC, Thornwood, NY, USA) and imaged at 20,000× to 63,000× magnification with an accelerating voltage of 60 kV (2 s exposure).

### Western blot

Protein from ultracentrifuge-precipitated exosomes was quantified by nanospectrophotometry and/or Qubit protein assay. Two micrograms of native exosomal protein from pooled CMEC and CMT samples was mixed with 4× Laemmli buffer heated at 95°C for 15 min. Proteins were resolved by SDS-PAGE on 4–20% precast polyacrylamide gels (Bio-Rad, Hercules, CA, USA) using the Precision Plus Protein Western C standards (Bio-Rad, Hercules, CA, USA) to determine the sizes of the bands, and then transferred to a nitrocellulose blotting membrane (LI-COR Biosciences, Lincoln, NE, USA). After electrophoresis, the fractions were electro-transferred to nitrocellulose membrane (Bio-Rad, Hercules, CA, USA) and blocked for 1 h. with Odyssey Blocking Buffer (LI-COR Biosciences, Lincoln, NE, USA). Membranes were incubated overnight at room temperature with 1:200 primary antibody CD9 Mouse-anti-Human (clone MM2/57, Bio-Rad AbD Serotec Inc., Hercules, CA, USA) in Odyssey Blocking Buffer (LI-COR Biosciences, Lincoln, NE, USA). Next the membranes were washed 3X for 10 min in 1X PBS in 0.1% Tween 20 (Sigma-Aldrich Corp., St. Louis, MO, USA). Next, secondary antibody IRDye Goat-anti-Mouse (LI-COR Biosciences, Lincoln, NE, USA) 1:10,000 dilution was incubated in Blocking Buffer (LI-COR Biosciences, Lincoln, NE, USA) for 1 h at 4 °C. Membranes were washed 3 times at room temperature with 1X PBS in 0.1% Tween 20 for 10 min. Fluorescent bands were visualized with Odyssey Near-Infrared Western Blot detection system in Image Studio (LI-COR Biosciences, Lincoln, NE, USA).

### RNA extraction & microRNA deep-sequencing

RNA was extracted from 5 mL of cell-free, serum-free conditioned media using the Norgen Biotek Urine/Cell Culture Exosomal RNA Isolation kit (Norgen Biotek, Thorold, ON, Canada) according to manufacturer instructions. After the lysis step, 10 pM final concentration synthetic miRVana cel-miR-39-3p mimic (Thermo Fisher Scientific, Waltham, MA, USA) was spiked into samples as an external control for technical variation. Extracted RNA was stored at − 80 °C until being shipped on dry ice to the Genomic Services Laboratory at the Hudson Alpha Institute for Biotechnology. Small RNA libraries were prepared for sequencing from total RNA from each sample using a NEBNext Small RNA Library Prep Set for Illumina (New England BioLabs Inc., Ipswich, MA, USA) according to the manufacturer’s protocol. Briefly, 3′ adapters were ligated to total input RNA followed by hybridization of multiplex SR RT primers and ligation of multiplex 5’ SR adapters. Reverse transcription (RT) was performed using ProtoScript II RT for 1 h at 50 °C. Immediately after the RT reaction, PCR amplification was performed for 15 cycles using LongAmp Taq 2X Master Mix. Illumina indexed primers were added to uniquely barcode each sample. Post-PCR material was purified using a QIAquick PCR purification kit (Qiagen Inc., Valencia, CA, USA). Post-PCR yield and concentration of the prepared libraries were assessed using a Qubit 2.0 Fluorometer (Invitrogen, Carlsbad, California, USA) and DNA 1000 chip on an Agilent 2100 Bioanalyzer (Applied Biosystems, Carlsbad, CA, USA), respectively. Size selection of small RNA was done using 3% dye free agarose gel cassettes on a Pippin Prep instrument (Sage Science Inc., Beverly, MA, USA). Post-size selection yield and concentration of the libraries were assessed using Qubit 2.0 Fluorometer and DNA High sensitivity chip on Agilent 2100 Bioanalyzer, respectively. Accurate quantification for sequencing applications was performed using the qPCR-based KAPA Biosystems Library Quantification kit (Kapa Biosystems, Inc., Woburn, MA, USA). Each library was diluted to a final concentration of 1.25 nM and pooled in equimolar ratios prior to clustering. Single End (SE) sequencing (50 bp) was performed to generate at least 15 million reads per sample on an Illumina HiSeq2500 sequencer (Illumina, Inc., San Diego, CA, USA).

Post processing of the sequencing reads from RNA-seq experiments from each sample was performed as per the Genomic Services Laboratory unique in-house pipeline. Briefly, quality control checks on raw sequence data from each sample was performed using FastQC (Babraham Bioinformatics, London, UK). Raw reads were imported on a commercial data analysis platform (AvadisNGS, Strand Scientifics, CA, USA). Adapter trimming was done to remove ligated adapter from 3′ ends of the sequenced reads with only one mismatch allowed, poorly aligned 3′ ends were also trimmed. Sequences shorter than 15 nucleotides length were excluded from further analysis. Trimmed reads with low qualities (base quality score less than 30, alignment score less than 95, mapping quality less than 40) were also removed. Filtered reads were then used to extract and count the small RNAs which were annotated using microRNAs from the miRBase release 20 database (http://www.mirbase.org/). Samples were subjected to quantification and active region quantification (AvadisNGS, Strand Scientifics, CA, USA). The quantification operation carries out measurement at both the gene level and at the active region level. Active region quantification considers only reads whose 5′ end matches the 5′ end of the mature miRNA annotation. Samples were then grouped by identifiers and the differential expression of each miRNA was calculated based on the fold change observed between different groups.

### qRT-PCR and data analysis

microRNA deep-sequencing results were validated by stem-loop quantitative RT-PCR (qPCR) for selected miRNA targets (selection process discussed in detail below). cDNA was created for each miRNA with a unique TaqMan™ stem-loop primer (Thermo Fisher Scientific, Waltham, MA, USA) with 1 ng RNA input using the TaqMan™ MicroRNA Reverse Transcription Kit (Thermo Fisher Scientific, Waltham, MA, USA) according to manufacturer instructions. A 1 μL cDNA product from the RT reaction was used as input for the qPCR reaction with TaqMan Universal Master Mix II no UNG (Thermo Fisher Scientific, Waltham, MA, USA), a specific 20X TaqMan microRNA assay for each target (Thermo Fisher Scientific, Waltham, MA, USA) and run in a BioRad CFX96 thermocycler (Bio-Rad, Hercules, CA, USA) according to the manufacturer protocol. Internal reference target miR-16 and external spike-in cel-miR-39 were used as control genes for normalization. Cq data was normalized using the ^2-ΔΔCq^ Livak method and presented as both log10 relative quantity for individual samples and fold change for the CMT group relative to the CMEC group [33]. miRNA that did not amplify were assigned a Cq number of 40 to allow calculation of normalized Cq values. These qPCR validation experiments were performed in triplicate and results were averaged and presented as mean ± SD.

### Selection of initial set of miRNAs

miRNAs of interest were selected from the set of differentially expressed genes. An initial set of 16 miRNAs were selected based on their expression profile and association with published studies in human and/or canine mammary neoplasia (cfa-miR-18a, cfa-miR-19a, cfa-miR-29c, cfa-miR-31, cfa-miR-34c, cfa-miR-105a, cfa-miR-181a, cfa-miR-206, cfa-miR-215, cfa-miR-345, cfa-miR-371, cfa-miR-495, cfa-miR-504, cfa-miR-615, cfa-miR-676, cfa-miR-1841). These were used for downstream bioinformatics analysis to identify putative gene targets for which enriched gene ontology terms and enriched biological pathways were identified.

### Gene target predictions

Target prediction for each miRNA was accomplished using the miRDB online resource and analysis platform (http://www.mirdb.org//). This tool was created in 2008 and was comprehensively updated recently when the complete set of miRNA sequences from the miR Base repository were downloaded along with the complete set of 3’UTR sequences contained in the NCBI RefSeq database. Furthermore, the miRDB target prediction algorithm, MirTarget, which was developed using support vector analysis of high throughput expression data, is capable of predicting conserved and non-conserved target genes via weighting target site conservation as a high priority, but not as an absolute requirement. miRDB scores predicted targets in a range from 50 to 100, with a higher score indicating a greater statistical confidence in the prediction. According to the FAQ on the miRDB website, “a predicted target with a score > 80 is most likely to be real.” Subsequently, target gene prediction was performed and scores greater than 80 were considered as representing the most confident gene predictions [34]. Gene targets for the complete set of 16 miRNAs were generated by selecting all gene targets having scores greater than 80 for each of the 16 miRNAs. The resulting set of all gene targets was filtered to remove redundant gene targets (i.e. gene targets that were associated with two or more different miRNAs).

### Gene ontology and KEGG pathway enrichment

The DAVID database for annotation, visualization and integrated discovery (version 6.8) was used to perform gene ontology enrichment analysis on sets of target genes using the gene symbol produced by the target prediction algorithm in miRDB. Canine gene symbols were uploaded into the DAVID database and the resulting sets of enriched gene ontology terms or KEGG pathways were identified [35].

### Statistical analysis

qRT-PCR relative quantity data were assessed for normality by visual inspection and Shapiro-Wilk test. Non-directional, non-parametric Mann-Whitney statistical testing was performed based on data that were not normally distributed. *p* < 0.05 was considered statistically significant for both RNAseq and qRT-PCR expression comparisons between groups. The DAVID gene ontology software provides both raw and Benjamini-corrected *p*-values, and a crude threshold of *p* < 0.06 was selected to screen for potentially relevant pathways [36].

## Results

### Characterization of extracellular vesicles

**Fig. 1:**
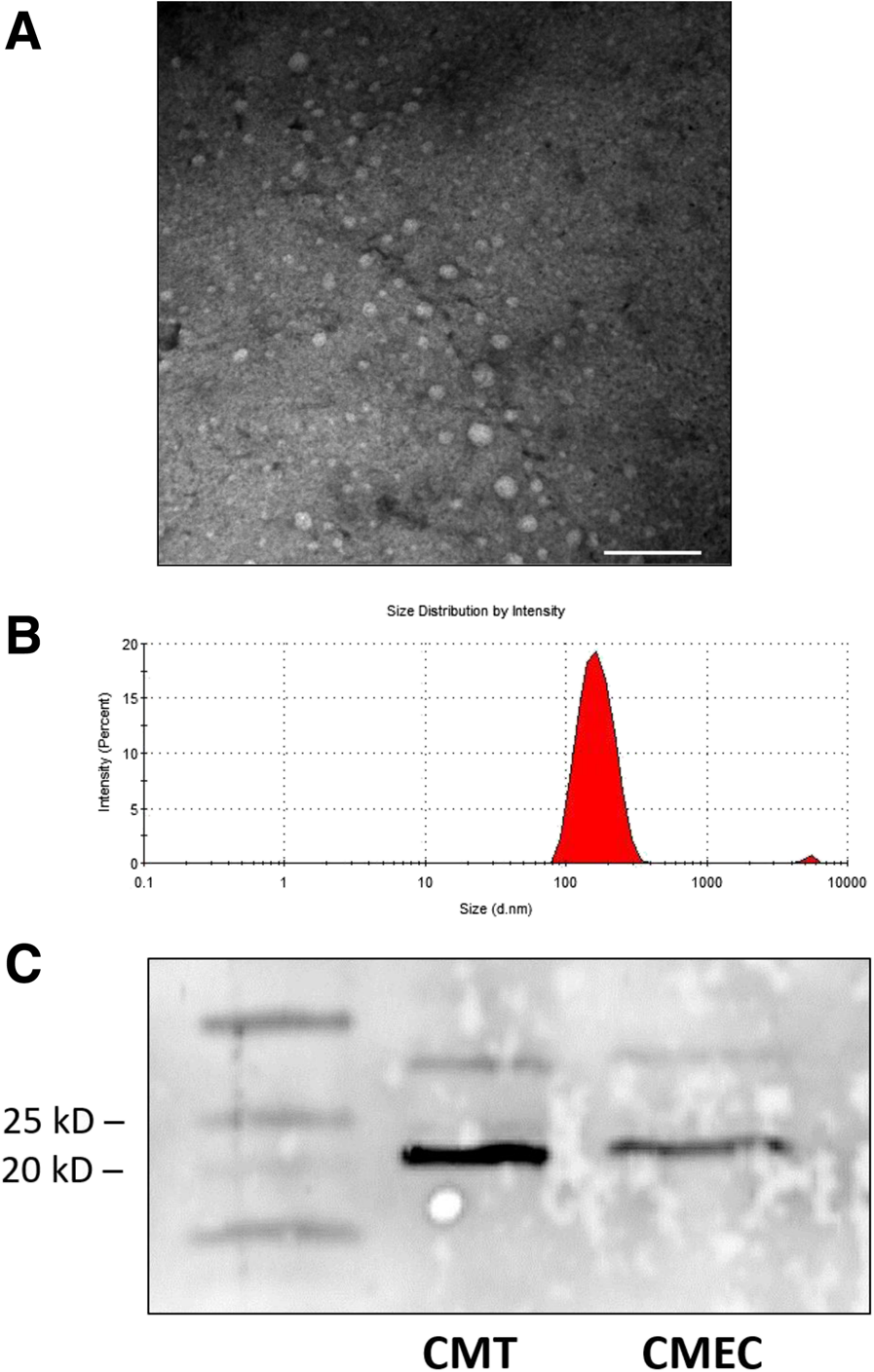
Characterization of putative exosomes. **a** Representative transmission electron micrograph (TEM) from ultracentrifuge-purified extracellular vesicles. Extracellular vesicles were irregularly round and varied widely in diameter from approximately 50–100 nm. Scale bar = 250 nm. **b** Representative intensity-weighted dynamic light scattering curve for ultracentrifuge-derived extracellular vesicle fractions. The mean intensity-weighted diameter of microvesicles varied from approximately 150–200 nm. The small peak between 1000 and 10,000 nm likely represents large polydisperse aggregates of particles. **c** Western blot of CD9 protein demonstrated both CMT and CMEC samples are positively immunoreactive for a protein approximately 20–25 kD in size. The CMT sample was more intensely positive despite the same total protein input, suggesting higher exosomal content in the CMT conditioned media

Ultrastructural evaluation of exosome-enriched supernatants using transmission electron microscopy confirmed the presence of variable numbers of irregularly round, occasionally cup-shaped vesicles ranging in size from approximately 60–120 nm in diameter (Fig. 1a). These vesicles occasionally formed variably-dense accumulations. Dynamic light scattering of this cell-free fraction showed a broad distribution of particle sizes with an average diameter of approximately 150 to 200 nm (Fig. 1b). The average protein concentrations in these cell-free fractions was 0.13 to 0.6 μg/μL. Putative exosome marker CD9 was detected in cell-free media from both CMEC and CMT cell lines by Western blotting (Fig. 1c). Our findings were consistent with having isolated exosome-like extracellular vesicles.

### microRNA profiling by small RNA deep-sequencing and qRT-PCR validation

**Fig. 2:**
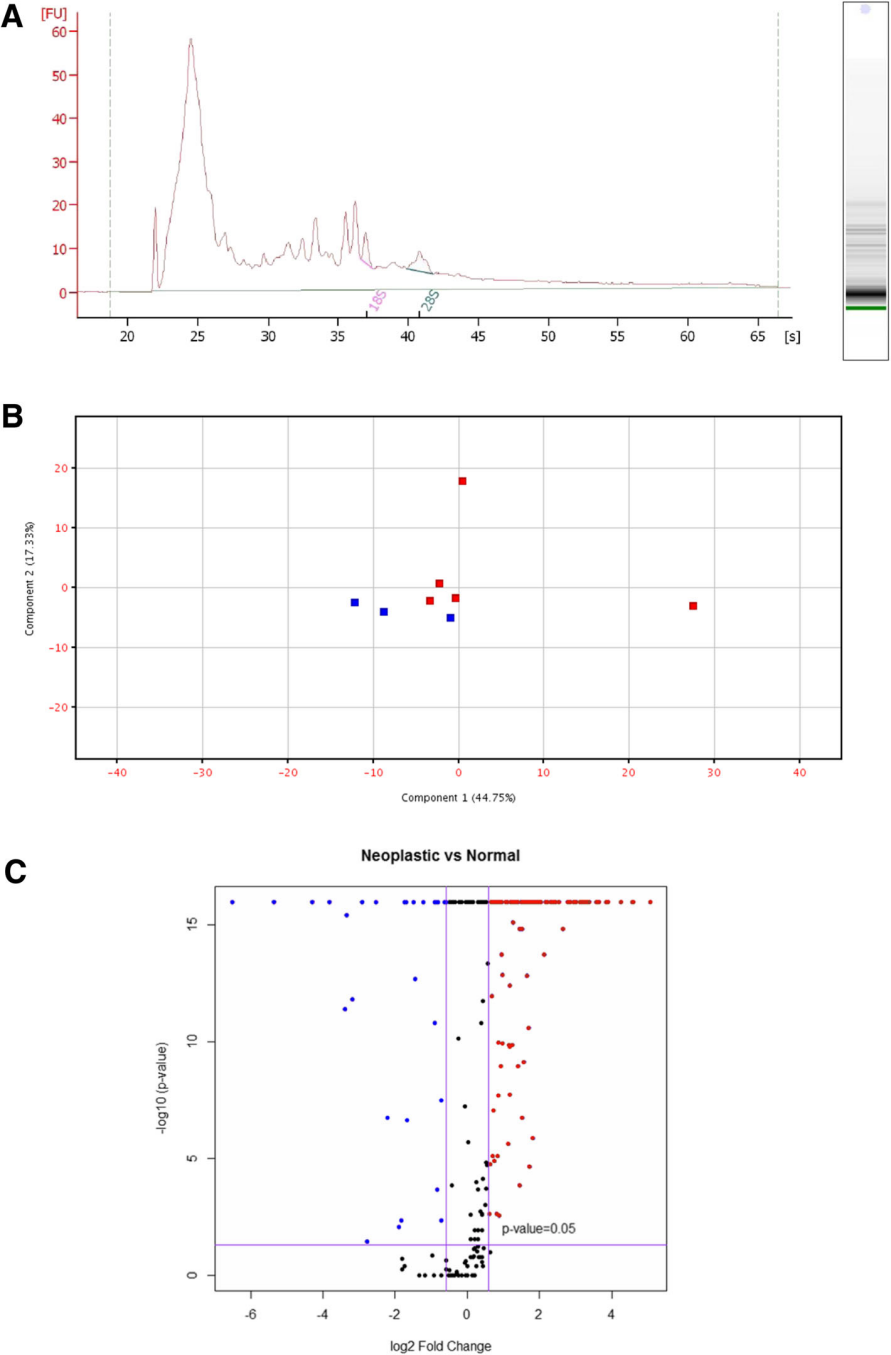
RNAseq profiling exosomal RNA. **a** RNA bioanalyzer fluorogram from CMT cell-free conditioned media showing a large proportion of the RNA is small in size (range likely to contain microRNAs). 18S and 28S markings denote location typical of rRNA peaks. **b** Principal Component Analysis (PCA) plot for microRNA profile by deep-sequencing comparing CMT (red) and CMEC (blue) group biological replicates. Distinct clustering between normal and neoplastic groups was observed. **c** Volcano plot showing up-regulated and down-regulated miRs. miRNAs in the upper right and upper left quadrants were statistically different between CMT and CMEC groups (*p* < 0.05). miRs identified in red were > 1.5-fold up-regulated in the CMT group relative to CMEC; miRs identified in blue were > 1.5-fold down-regulated in the CMT group relative to CMEC

The RNA bioanalyzer profiles were typical of exosomal samples, skewing heavily towards small RNA (~ 20–200 nucleotides), with minimal detection of rRNA (Fig. 2a). The normalized miRNA in reads per million for all samples are provided in Additional file 1: Table S1. Three hundred thirty-eight unique miRs were detected in the cell-free RNA fractions from CMEC and CMT samples. In a principal component analysis of the miRNA from all eight samples the CMEC and CMT samples clustered into two separate groups, although the CMT group had two significant outliers (Fig. 2b).

Volcano plot analysis illustrates that numerous miRs were significantly over- and under-expressed by CMT exosomes relative to CMEC (Fig. 2c). Using criteria of *p* < 0.05 and a fold-change ≥ ± 1.5-fold change, there were 170 differentially expressed miRs between CMT and CMEC groups (Table 1). Removing isoform miRs from different chromosomal locations yielded 145 unique differentially expressed miRs, with 118 miRs upregulated and 27 miRs downregulated in CMT as compared to CMEC.

**Table 1:** Complete list of statistically significant, differentially expressed miRNAs. Fold-change and direction of regulation refer to the CMT group versus the CMEC group expression

miR	Gene ID	Fold-change	Regulation	*p*-value	*p*-value (corrected)
miR-9	MI0008125_1	33.36	up	0.00E + 00	0.00E + 00
miR-9	MI0008081_1	33.36	up	0.00E + 00	0.00E + 00
miR-9	MI0008086_1	33.36	up	0.00E + 00	0.00E + 00
miR-122	MI0008015_1	24.08	up	0.00E + 00	0.00E + 00
miR-183	MI0008017_1	23.75	up	0.00E + 00	0.00E + 00
miR-182	MI0010336_1	18.90	up	0.00E + 00	0.00E + 00
miR-106b	MI0008109_1	14.72	up	0.00E + 00	0.00E + 00
mIR-31	MI0007994_1	14.32	up	0.00E + 00	0.00E + 00
miR-429	MI0001644_1	12.47	up	0.00E + 00	0.00E + 00
miR-203	MI0010363_1	11.79	up	0.00E + 00	0.00E + 00
miR-18a	MI0010324_1	10.34	up	0.00E + 00	0.00E + 00
miR-146a	MI0008094_1	10.13	up	0.00E + 00	0.00E + 00
miR-181c	MI0008034_1	9.99	up	0.00E + 00	0.00E + 00
miR-96	MI0010356_1	9.65	up	0.00E + 00	0.00E + 00
miR-135b	MI0010334_1	9.25	up	0.00E + 00	0.00E + 00
miR-181b	MI0008153_1	8.75	up	0.00E + 00	0.00E + 00
miR-181b	MI0008127_1	8.75	up	0.00E + 00	0.00E + 00
miR-196a	MI0010360_1	8.69	up	0.00E + 00	0.00E + 00
miR-200b	MI0010361_1	8.11	up	0.00E + 00	0.00E + 00
miR-181a	MI0008152_1	7.78	up	0.00E + 00	0.00E + 00
miR-181a	MI0008126_1	7.62	up	0.00E + 00	0.00E + 00
miR-15b	MI0008083_1	7.26	up	0.00E + 00	0.00E + 00
miR-371	MI0007996_1	7.11	up	0.00E + 00	0.00E + 00
miR-371	MI0007996_2_1	7.11	up	0.00E + 00	0.00E + 00
miR-363	MI0008176_1	7.01	up	0.00E + 00	0.00E + 00
miR-103	MI0010357_1	6.82	up	0.00E + 00	0.00E + 00
miR-1841	MI0008096_1	6.28	up	4.44E-16	6.49E-16
miR-30b	MI0008013_1	5.82	up	0.00E + 00	0.00E + 00
miR-200a	MI0010362_1	5.46	up	0.00E + 00	0.00E + 00
miR-34c	MI0008106_1	5.43	up	0.00E + 00	0.00E + 00
miR-146b	MI0008073_1	5.35	up	0.00E + 00	0.00E + 00
miR-331	MI0010394_1	5.24	up	0.00E + 00	0.00E + 00
miR-147	MI0010371_1	5.24	up	0.00E + 00	0.00E + 00
miR-155	MI0008078_1	5.10	up	0.00E + 00	0.00E + 00
miR-20a	MI0008052_1	4.99	up	0.00E + 00	0.00E + 00
miR-19b	MI0008054_1	4.96	up	0.00E + 00	0.00E + 00
miR-19b	MI0008174_1	4.96	up	0.00E + 00	0.00E + 00
miR-107	MI0008072_1	4.92	up	0.00E + 00	0.00E + 00
miR-181d	MI0008035_1	4.66	up	0.00E + 00	0.00E + 00
miR-200c	MI0008070_1	4.53	up	0.00E + 00	0.00E + 00
miR-345	MI0008129_1	4.41	up	5.33E-15	7.54E-15
miR-130a	MI0008029_1	4.08	up	0.00E + 00	0.00E + 00
miR-29c	MI0008122_1	4.06	up	0.00E + 00	0.00E + 00
miR-29c	MI0015960_1	4.06	up	0.00E + 00	0.00E + 00
miR-15a	MI0008048_1	4.03	up	0.00E + 00	0.00E + 00
miR-19a	MI0008051_1	3.84	up	0.00E + 00	0.00E + 00
miR-16	MI0008084_1	3.71	up	0.00E + 00	0.00E + 00
miR-93	MI0008110_1	3.56	up	0.00E + 00	0.00E + 00
miR-1343	MI0027953_1	3.53	up	0.00E + 00	0.00E + 00
miR-7	MI0010330_1	3.51	up	0.00E + 00	0.00E + 00
miR-874	MI0010429_1	3.51	up	4.76E-07	5.65E-07
miR-103	MI0008098_1	3.48	up	0.00E + 00	0.00E + 00
miR-7	MI0008033_1	3.46	up	0.00E + 00	0.00E + 00
miR-27a	MI0008040_1	3.46	up	0.00E + 00	0.00E + 00
miR-1839	MI0008087_1	3.43	up	0.00E + 00	0.00E + 00
miR-196a	MI0008068_1	3.43	up	0.00E + 00	0.00E + 00
miR-20b	MI0010322_1	3.41	up	0.00E + 00	0.00E + 00
miR-660	MI0008186_1	3.39	up	0.00E + 00	0.00E + 00
miR-215	MI0010343_1	3.29	up	8.72E-06	9.96E-06
miR-186	MI0008108_1	3.27	up	0.00E + 00	0.00E + 00
miR-495	MI0008140_1	3.25	up	8.10E-12	1.07E-11
miR-339	MI0008115_1	3.25	up	0.00E + 00	0.00E + 00
miR-421	MI0008181_1	3.16	up	4.40E-14	6.13E-14
miR-16	MI0008049_1	3.14	up	0.00E + 00	0.00E + 00
miR-27b	MI0008009_1	3.12	up	0.00E + 00	0.00E + 00
miR-543	MI0008139_1	3.10	up	0.00E + 00	0.00E + 00
miR-205	MI0010340_1	3.05	up	0.00E + 00	0.00E + 00
miR-29a	MI0008022_1	3.03	up	0.00E + 00	0.00E + 00
miR-192	MI0008031_1	3.03	up	0.00E + 00	0.00E + 00
miR-340	MI0010391_1	3.01	up	0.00E + 00	0.00E + 00
miR-503	MI0008170_1	2.95	up	0.00E + 00	0.00E + 00
miR-1296	MI0028014_1	2.93	up	2.45E-10	3.11E-10
miR-499	MI0008059_1	2.91	up	0.00E + 00	0.00E + 00
miR-7	MI0008085_1	2.89	up	0.00E + 00	0.00E + 00
miR-486	MI0008027_2	2.87	up	4.44E-16	6.49E-16
miR-212	MI0008155_1	2.87	up	6.12E-08	7.41E-08
miR-30d	MI0008012_1	2.81	up	0.00E + 00	0.00E + 00
miR-615	MI0010419_1	2.73	up	5.27E-05	5.99E-05
miR-184	MI0010337_1	2.71	up	4.44E-16	6.49E-16
miR-130b	MI0008064_1	2.66	up	3.81E-10	4.77E-10
miR-590	MI0008114_1	2.64	up	0.00E + 00	0.00E + 00
miR-23a	MI0008039_1	2.58	up	0.00E + 00	0.00E + 00
miR-185	MI0008065_1	2.55	up	0.00E + 00	0.00E + 00
miR-335	MI0008020_1	2.53	up	0.00E + 00	0.00E + 00
miR-22	MI0008157_1	2.51	up	0.00E + 00	0.00E + 00
miR-125b	MI0008077_1	2.50	up	0.00E + 00	0.00E + 00
miR-1307	MI0008071_1	2.46	up	0.00E + 00	0.00E + 00
miR-1301	MI0027930_1	2.43	up	2.22E-16	3.32E-16
miR-375	MI0010368_1	2.38	up	4.60E-11	5.97E-11
miR-23b	MI0008008_1	2.31	up	0.00E + 00	0.00E + 00
miR-125b	MI0008103_1	2.30	up	0.00E + 00	0.00E + 00
miR-324	MI0010395_1	2.27	up	6.64E-09	8.25E-09
miR-126	MI0008154_1	2.25	up	1.22E-13	1.68E-13
miR-542	MI0008171_1	2.25	up	5.30E-11	6.77E-11
miR-6529	MI0027868_1	2.23	up	4.73E-11	6.08E-11
miR-323	MI0008137_1	2.19	up	8.21E-07	9.69E-07
miR-365	MI0001657_1	2.17	up	0.00E + 00	0.00E + 00
miR-365	MI0001647_1	2.16	up	0.00E + 00	0.00E + 00
miR-92a	MI0008055_1	2.16	up	0.00E + 00	0.00E + 00
miR-374b	MI0008180_1	2.16	up	0.00E + 00	0.00E + 00
miR-1306	MI0008066_1	2.13	up	0.00E + 00	0.00E + 00
miR-29b	MI0008121_1	2.11	up	0.00E + 00	0.00E + 00
miR-502	MI0008187_1	2.10	up	0.00E + 00	0.00E + 00
miR-92a	MI0008175_1	2.10	up	0.00E + 00	0.00E + 00
miR-410	MI0008149_1	1.97	up	3.84E-11	5.02E-11
miR-101	MI0008107_1	1.95	up	0.00E + 00	0.00E + 00
miR-32	MI0007992_1	1.95	up	4.17E-14	5.86E-14
miR-376a	MI0008141_1	1.93	up	5.33E-15	7.54E-15
miR-376a	MI0008142_1	1.93	up	5.33E-15	7.54E-15
miR-376a	MI0008143_1	1.93	up	5.33E-15	7.54E-15
miR-381	MI0010390_1	1.92	up	0.00E + 00	0.00E + 00
miR-454	MI0010426_1	1.91	up	3.81E-10	4.77E-10
miR-8859a	MI0027950_1	1.89	up	0.00E + 00	0.00E + 00
miR-140	MI0008100_1	1.88	up	0.00E + 00	0.00E + 00
miR-425	MI0008038_1	1.87	up	0.00E + 00	0.00E + 00
miR-500	MI0008185_1	1.85	up	0.00E + 00	0.00E + 00
miR-2387	MI0027966_1	1.84	up	1.14E-03	1.26E-03
miR-30a	MI0008000_1	1.83	up	0.00E + 00	0.00E + 00
miR-758	MI0010424_1	1.82	up	3.43E-11	4.51E-11
miR-329b	MI0010398_1	1.82	up	6.91E-09	8.54E-09
miR-382	MI0008145_1	1.80	up	2.95E-06	3.44E-06
miR-24	MI0008010_1	1.78	up	0.00E + 00	0.00E + 00
miR-144	MI0008158_1	1.75	up	9.22E-04	1.03E-03
miR-24	MI0008041_1	1.75	up	0.00E + 00	0.00E + 00
miR-101	MI0007995_1	1.73	up	0.00E + 00	0.00E + 00
miR-301b	MI0010349_1	1.68	up	4.74E-06	5.49E-06
miR-148b	MI0008069_1	1.68	up	0.00E + 00	0.00E + 00
miR-485	MI0008146_1	1.65	up	2.96E-08	3.60E-08
miR-125a	MI0008005_1	1.65	up	0.00E + 00	0.00E + 00
miR-379	MI0008134_1	1.62	up	0.00E + 00	0.00E + 00
let-7 g	MI0008036_1	1.62	up	0.00E + 00	0.00E + 00
miR-18b	MI0010323_1	1.62	up	2.79E-06	3.27E-06
miR-21	MI0008165_1	1.61	up	0.00E + 00	0.00E + 00
miR-505	MI0010407_1	1.59	up	3.46E-13	4.71E-13
miR-494	MI0010404_1	1.57	up	0.00E + 00	0.00E + 00
miR-380	MI0008136_1	1.55	up	6.32E-06	7.27E-06
miR-138a	MI0008056_1	1.52	up	9.49E-04	1.06E-03
miR-26a	MI0008058_1	−1.51	down	0.00E + 00	0.00E + 00
miR-26a	MI0007990_1	−1.51	down	0.00E + 00	0.00E + 00
miR-222	MI0010346_1	−1.56	down	0.00E + 00	0.00E + 00
miR-8884	MI0027983_1	−1.65	down	1.15E-08	1.41E-08
miR-490	MI0010372_1	−1.66	down	1.82E-03	1.99E-03
miR-30c	MI0008024_1	−1.77	down	0.00E + 00	0.00E + 00
miR-30c	MI0008001_1	−1.77	down	0.00E + 00	0.00E + 00
miR-99a	MI0008102_1	−1.79	down	0.00E + 00	0.00E + 00
miR-99a	MI0008075_1	−1.79	down	0.00E + 00	0.00E + 00
miR-127	MI0008132_1	−1.80	down	8.70E-05	9.83E-05
miR-30e	MI0008023_1	−1.87	down	5.28E-12	7.04E-12
miR-889	MI0027984_1	−1.87	down	0.00E + 00	0.00E + 00
miR-374a	MI0008179_1	−2.33	down	0.00E + 00	0.00E + 00
miR-455	MI0007999_1	−2.71	down	6.24E-14	8.63E-14
miR-145	MI0010359_1	−2.81	down	0.00E + 00	0.00E + 00
miR-574	MI0008080_1	−3.18	down	8.45E-08	1.01E-07
miR-152	MI0008162_1	−3.25	down	0.00E + 00	0.00E + 00
miR-148a	MI0008018_1	−3.36	down	0.00E + 00	0.00E + 00
miR-8865	MI0027958_1	−3.53	down	1.82E-03	1.99E-03
miR-676	MI0008188_1	−3.71	down	3.65E-03	3.97E-03
miR-105a	MI0010377_1	−4.63	down	6.60E-08	7.94E-08
miR-143	MI0008092_1	−5.78	down	0.00E + 00	0.00E + 00
miR-196b	MI0008016_1	−6.92	down	1.64E-02	1.77E-02
miR-1	MI0008118_1	−7.52	down	0.00E + 00	0.00E + 00
miR-1	MI0008060_1	−7.52	down	0.00E + 00	0.00E + 00
miR-214	MI0010342_1	−9.13	down	4.89E-13	6.61E-13
miR-504	MI0010406_1	−10.06	down	1.11E-16	1.67E-16
miR-383	MI0008026_1	−10.41	down	1.28E-12	1.72E-12
miR-199	MI0008151_1	−14.03	down	0.00E + 00	0.00E + 00
miR-199	MI0008042_1	−14.03	down	0.00E + 00	0.00E + 00
miR-199	MI0008124_1	−19.70	down	0.00E + 00	0.00E + 00
miR-10a	MI0008161_1	−41.07	down	0.00E + 00	0.00E + 00
miR-206	MI0008002_1	−91.77	down	0.00E + 00	0.00E + 00

**Fig. 3:**
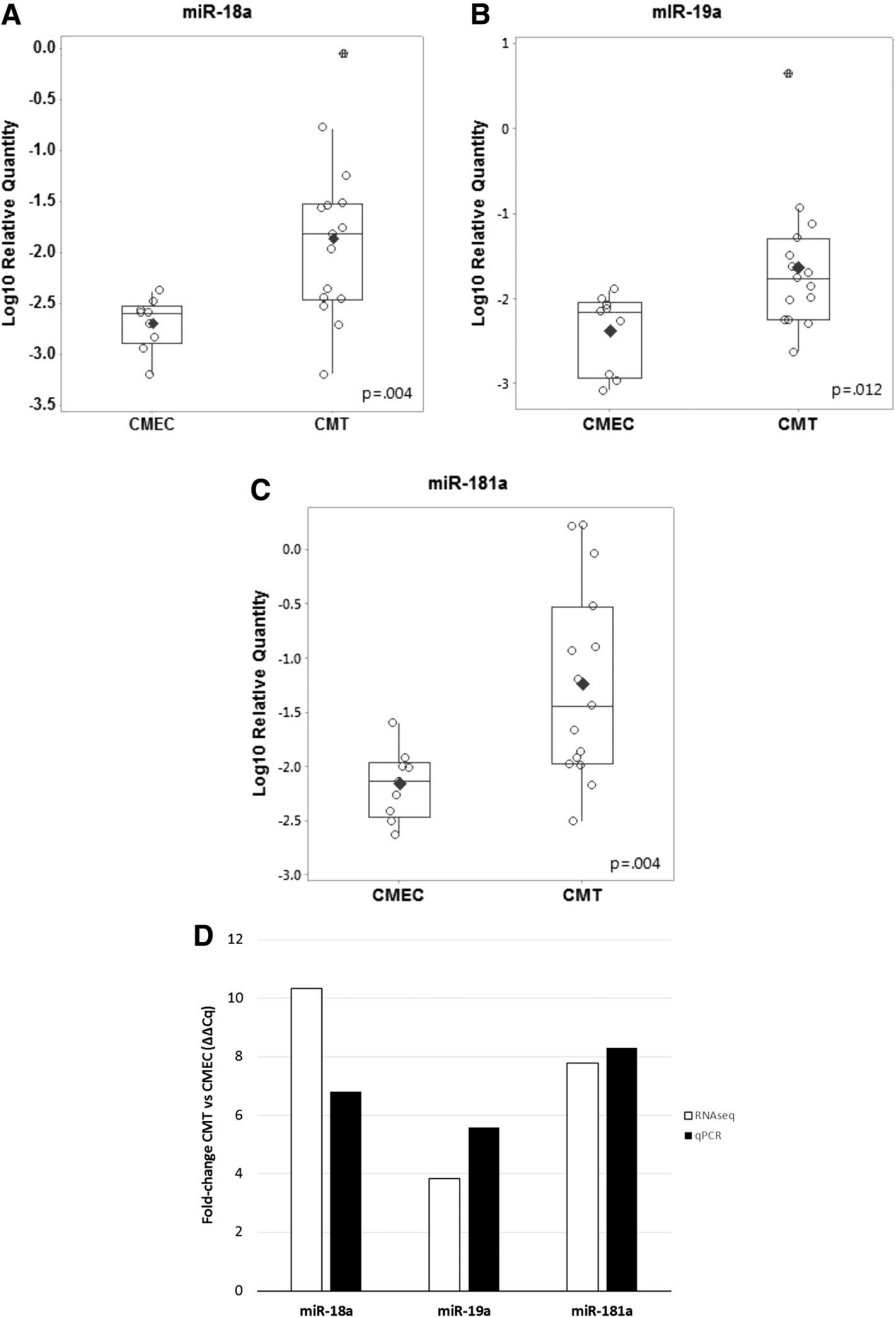
qRT-PCR validation of RNAseq data. **a-c** Relative quantification (log10) for selected validation targets miR-18a (**a**), miR-19a (**b**), and miR-181a (**c**). Relative quantification was calculated for each biological replicate according to the eq. 2^-ΔCq^, with cel-miR-39 as spike-in exogenous control and miR-16 as an endogenous control; experiments were performed in triplicate from cell culture to RNA extraction, cDNA synthesis and qRT-PCR. Data were not normally distributed and compared by non-parametric, non-directional Mann-Whitney test. *p* < 0.05 was considered statistically significant. The black horizontal line represents the group mean and the vertical “whiskers” represent ±1 SD. **d** Comparison of fold-change between microRNA deep-sequencing and manual stem-loop qRT-PCR assays for selected targets miR-18a, miR-19a, and miR-181a. Data were normalized using the 2^-ΔΔCq^ method. The average group Cq for cfa-miR-16 (endogenous control) and cel-miR-39 (exogenous spike-in control) were used as housekeeping genes for normalization. White bars represent relative fold-change for RNAseq data, black bars represent fold-change for qRT-PCR (3 experimental replicates)

Three of the significantly upregulated miRs (miR-18a, miR-19a, and miR-181a) were selected for qRT-PCR validation. The average relative quantities (log10 2^^-ΔCq^) for cfa-miR-18a, cfa-miR-19a, and cfa-miR-181a for CMEC and CMT groups were significantly higher in the CMT group compared to CMEC (Fig. 3a-c). Each of these three miRs showed very similar fold-change between CMT and CMEC for both small RNA deep-sequencing and qRT-PCR assays (Fig. 3d). Table 2 compares fold-change and *p*-values for RNAseq and qRT-PCR data.

**Table 2:** Comparison of microRNA expression by RNAseq and qRT-PCR

microRNA	RNAseq		qRT-PCR	
	Fold-change	*p*-value	Fold-change	*p*-value
miR-18a	10.34	0	6.82	0.004
miR-19a	3.84	0	5.58	0.012
miR-181a	7.70	0	8.30	0.004

### In silico analysis of microRNA targets

**Fig. 4:**
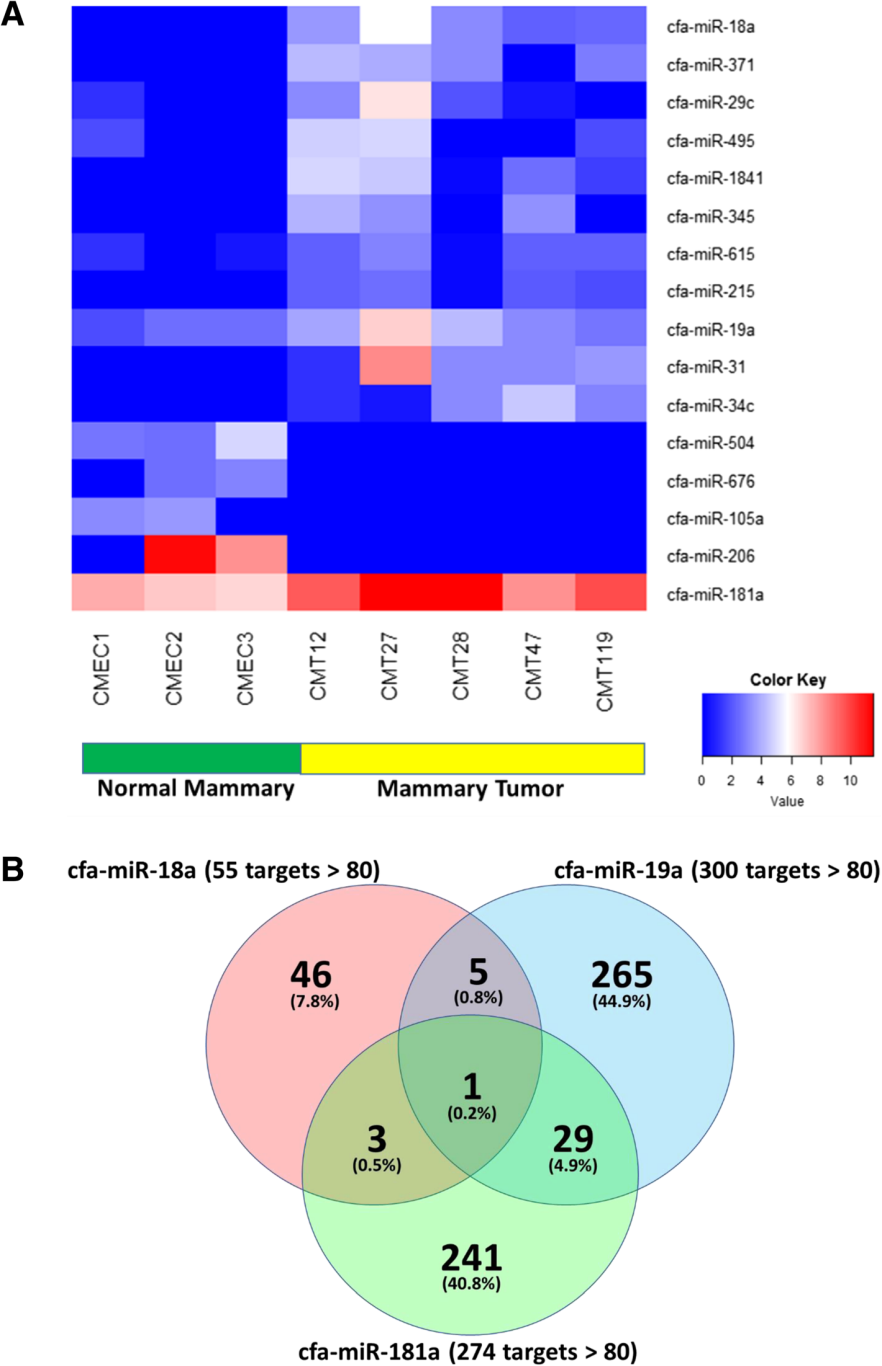
Overlap of target genes in the miRNA subset. **a** Supervised absolute expression heat map for 16 canine miRNAs in CMEC vs. CMT exosomal RNA samples. Three biological replicates (CMEC1, CMEC2, and CMEC3) corresponding to normal mammary tissue exhibit relatively low levels of expression for the first eleven miRNAs while the last five miRNAs exhibit considerably higher levels of expression. The pattern is reversed in the mammary tumor samples (CMT12, CMT27, CMT28, CMT47, and CMT119) as visualized in the right side of the heat map. Because of the dichotomous pattern of expression across the control and mammary samples, these miRNAs may represent valuable candidates for clinically relevant biomarkers. **b** A miRNA subset was selected from among the 16 miRNAs (**a**). Overlap of target genes is indicated by both numbers of target genes and percentage of total genes (sum of each miRNA’s target gene set). Cfa-miR-18a represents an miRNA with relatively low expression in normal mammary tissue and higher expression in mammary tumor samples. Similarly, cfa-miR-19a also exhibits low expression in normal mammary samples and higher expression in mammary tumor samples. In contrast, cfa-miR-181a exhibits considerably higher expression in the normal mammary tissue compared to 18a and 19a. Additionally, 181a expression in mammary tumor samples is greater than any other miRNA among the 16 miRNAs represented in (**a**)

A set of 16 differentially expressed miRNAs from this data set were selected for in silico analysis of canine predicted miR targets (Fig. 4a). The number of predicted genes identified per miRNA ranged from 124 to 751 genes for total predictions and from 24 to 300 genes for predictions having a score greater than 80 (Table 3). To gain an appreciation for the types of biological processes associated with these 16 miRNAs, the complete set of gene targets for all 16 miRNAs was used for a gene ontology biological process enrichment analysis (Additional file 2: Table S2).

**Table 3:** Number of Predicted Gene Targets for selected miRNAs of biological interest

miRNA	Total Targets Predicted	Targets with Score > 80
cfa-miR-18a	181	55
cfa-miR-19a	646	300
cfa-miR-29c	536	208
cfa-miR-31	359	98
cfa-miR-34c	420	154
cfa-miR-105a	362	105
cfa-miR-181a	694	274
cfa-miR-206	358	133
cfa-miR-215	124	24
cfa-miR-345	215	58
cfa-miR-371	550	164
cfa-miR-495	729	263
cfa-miR-504	176	43
cfa-miR-615	163	39
cfa-miR-676	552	182
cfa-miR-1841	751	219

The complete set of all 2323 miRNA target genes with scores greater than 80 were selected. Following removal of redundant target genes in the list, we identified a set of 1849 unique target genes. The filtered list was used in the DAVID database and, of the 1849 gene symbols uploaded, 1819 were successfully mapped to canine genes using the DAVID gene list manager. In contrast, the gene list manager identified 1737 of the 1849 gene symbols as human genes.

### Gene ontology enrichment across all gene targets

A total of 145 gene ontology terms were identified as enriched, with 85 having *p*-values less than or equal to 0.06. Although we considered GO annotations with *p*-value < 0.06, we focused on those with *p*-value < 0.05 (Table 4). The complete set of all identified terms for the combined target genes is included in Additional file 3: Table S3, along with the list of specific genes associated with each enriched GO term. A set of 34 enriched terms are also included (Table 4). These terms provide some insight into the cellular role of the 16 miRNAs.

**Table 4:** Enriched gene ontology (GO) biological process enriched terms associated with combined set of predicted target genes

GO biological process term	Number	Percent	*p*-Value	Benjamini
positive regulation of transcription from RNA polymerase II promoter	87	4.8	5.10E-05	1.60E-01
ubiquitin-dependent protein catabolic process	23	1.3	5.70E-04	6.30E-01
G1/S transition of mitotic cell cycle	13	0.7	8.50E-04	6.20E-01
positive regulation of glucose import in response to insulin stimulus	6	0.3	4.10E-03	8.70E-01
negative regulation of canonical Wnt signaling pathway	18	1	5.30E-03	9.00E-01
regulation of establishment of cell polarity	6	0.3	6.40E-03	9.10E-01
regulation of cell morphogenesis	7	0.4	7.30E-03	8.80E-01
protein autophosphorylation	22	1.2	7.70E-03	8.70E-01
vesicle fusion	12	0.7	7.90E-03	8.60E-01
negative regulation of apoptotic process	35	1.9	8.90E-03	8.70E-01
regulation of small GTPase mediated signal transduction	6	0.3	9.50E-03	8.70E-01
regulation of mRNA stability	6	0.3	9.50E-03	8.70E-01
TOR signaling	6	0.3	9.50E-03	8.70E-01
intrinsic apoptotic signaling pathway by p53 class mediator	7	0.4	9.90E-03	8.70E-01
negative regulation of extrinsic apoptotic signaling pathway	9	0.5	1.10E-02	8.60E-01
histone ubiquitination	5	0.3	1.10E-02	8.40E-01
neuronal stem cell population maintenance	7	0.4	1.30E-02	8.40E-01
chromatin remodeling	12	0.7	1.50E-02	8.60E-01
response to hypoxia	15	0.8	1.60E-02	8.70E-01
mRNA splice site selection	5	0.3	1.80E-02	8.70E-01
miRNA mediated inhibition of translation	5	0.3	1.80E-02	8.70E-01
regulation of gene expression	14	0.8	2.20E-02	8.80E-01
positive regulation of cell-substrate adhesion	8	0.4	2.80E-02	9.00E-01
histone H3-K9 trimethylation	3	0.2	3.30E-02	9.20E-01
histone H2A ubiquitination	3	0.2	3.30E-02	9.20E-01
polarized epithelial cell differentiation	3	0.2	3.30E-02	9.20E-01
regulation of blood coagulation	5	0.3	3.50E-02	9.20E-01
positive regulation of cell proliferation	34	1.9	3.80E-02	9.30E-01
positive regulation of erythrocyte differentiation	6	0.3	4.00E-02	9.30E-01
negative regulation of cell migration	13	0.7	4.30E-02	9.40E-01
histone H3-K4 trimethylation	5	0.3	4.60E-02	9.50E-01
positive regulation of apoptotic process	21	1.2	4.60E-02	9.40E-01
stem cell population maintenance	9	0.5	5.00E-02	9.50E-01
cell migration	19	1.0	5.32E-02	9.50E-01

Among the enriched biological processes are a number implicated in cell division including: G1/S transition of mitotic cell cycle; negative regulation of apoptotic process; intrinsic apoptotic signaling pathway by p53 class mediator; positive regulation of cell proliferation and positive regulation of apoptotic process. Additional terms, associated with specific cellular processes involved in development and differentiation were also identified such as: regulation of establishment of cell polarity; regulation of cell morphogenesis; neuronal stem cell population maintenance; polarized epithelial cell differentiation; positive regulation of erythrocyte differentiation; and positive regulation of cell-substrate adhesion. Finally, terms associated with chromatin remodeling were present including: histone ubiquitination; chromatin remodeling; histone H3-K9 trimethylation; histone H2A ubiquitination; and histone H3-K4 trimethylation.

### Small subset of miRNAs having potential for clinical biomarker relevance

Based on the initial gene ontology enrichment analysis of all target genes associated with the set of 16 miRNAs, a choice was made to select a relatively small subset that might be suitable candidates for downstream clinical biomarker applications. The overarching goal in selecting the miRNA subset was focused upon (1) maximizing the representation of target genes associated with the set of enriched gene ontology terms while simultaneously (2) minimizing the number of miRNAs selected. Analysis of target gene representation associated with enriched gene ontology terms identified three miRNAs: cfa-miR-18a, cfa-miR-19a, and cfa-miR-181a. Together, these three miRNAs contain target genes for all but one enriched gene ontology term listed in Table 3 (histone H3-K9 trimethylation). Moreover, the overlap of target genes between these miRNAs was less than 7% (Fig. 4b).

### Functional annotation of miRNAs cfa-miR-18a, cfa-miR-19a, and cfa-miR-181a

The target genes predicted for cfa-miR-18a were used to generate functional annotation using the DAVID database. The set of 53 predicted target genes were submitted to the DAVID database to identify relevant functional annotation. Upon submission, 52 genes were successfully mapped. Gene enrichment analysis was performed using the biological process terms (GOTERM_BP_ALL) (Table 4). “Epithelial development” was identified as being enriched with *p*-value = 0.0192 in association with the ESR1, FRS2, HIF1A, and PDE4D genes. The terms “mammary gland lobule development” and “mammary gland alveolus development” were both associated with the ESR1 gene and the HIF1A gene (*p*-value = 0.052). The pathway “proteoglycans in cancer” was identified as an enriched KEGG pathway with *p*-value = 0.0099. The genes associated with this pathway were ESR1, HIF1A, FRS2, SDC4.

Analysis of cfa-miR-19a was based on the set of 300 predicted target genes, 299 of which were successfully mapped to canine genes using the DAVID gene list manager. Gene ontology biological process terms enriched included endothelial cell apoptotic process associated with BMPR2 and HIPK1 (*p*-value = 0.052). Additionally, the term cell proliferation was associated with E2F8, LRP2, MDM4, APPL1, and ANXA7 (*p*-value = 0.06). Enriched pathways obtained from KEGG pathway enrichment analysis included: renal cell carcinoma associated with RAP1A, RAP1B, PAK6, PIK3CB and PIK3R3 (*p*-value = 0.011); cGMP-PKG signaling pathway with ATF2, CACNA1C, MEF2A, NFATC2, PIK3CB, PDE5, PIK3R3, and SLC25A6 (*p*-value = 0.0015); MAPK signaling pathway in association with RAP1A, RAP1B, RAPGEF2, TAOK1, ATF2, CACNA1C, MAPK8, MAP3K12 and RPS6KA5 (*p*-value = 0.017); FoxO signaling pathway through an association with FBXo32, CCND2, PIK3CB, PIK3R3, and S1PR1 (*p*-value = 0.003); and colorectal cancer with the genes APPL1, MAPK8, PIK3CB, and PIK3R3 (*p*-value = 0.06).

Similar analysis of the 274 gene targets of cfa-miR-181a resulted in the successful mapping of 261 canine genes in the DAVID database. The analysis identified enriched gene ontology biological process terms: Microtubule Anchoring in association with FOPNL, GCC2 and CLASP1 (*p*-value = 0.014); Positive Regulation of Transcription from RNA Polymerase II Promoter in association with DDX3, INO80, KLF15, LMO1, RORA, TAF9B, ATXN7, CDON, CCNK, HMGB2, IL1A, KMT2A, PROX1, RPS6KA3, and THRB (*p*-value = 0.053). KEGG pathway enrichment analysis identified Protein Processing in Endoplasmic Reticulum with DNAJC3, SEC24A, SEC24C, SEL1, ATXN3, HSP90B1, and MBTPS2 (*p*-value = 0.017); Glycerophospholipid Metabolism in association with DGKQ, ETNK1, GPD1L, LCLAT1, and MBOAT1 (*p*-value = 0.026); and Phosphatidylinositol Signaling System in association with DGKQ, PPIP5K2, INPP4A, PI4K2B, and PLCB1 (*p*-value = 0.031). A full list of gene ontology processes predicted to be targeted by miR-18a, miR-19a and miR-181a is provided (Table 5).

**Table 5:** Predicted target genes in representative set of enriched gene ontology biological process terms

Enriched Term	Gene Ontology Id	Target Genes (within all 16 miRNAs)	Targets in 3 miRNA Subset
G1/S transition of mitotic cell cycle	GO:0000082	CCNE2, ACVR1B, EIF4E, CACUL1, CAMK2G, CAMK2D, USP37, RANBP1, RPS6KB1, PPP3CA, PHF8, LATS2, RBBP8	19a: CACUL1, RBBP81 181a: RPS6KB1
regulation of establishment of cell polarity	GO:2000114	ROCK1, GATA3, KRIT1, RICTOR, ARFGEF1, KANK1	19a: ARFGEF1 181: KANK1
negative regulation of apoptotic process	GO:0043066	FKBP8, TAF9B, NAA15, FOXO1, TP63, CITED2, SETX, PTK2, CASP3, DAB2, PRKAA1, RARB, HSPA5, AGO4, DNAJC3, KLHL20, CDK1, PDCD10, ADAMTS20, ZNF830, CBL, ASIC2, IGF1, RHBDD1, UBE2B, ASCL1, HSP90B1, GSK3B, HIPK3, VEGFA, ARF4, MAPK8, MDM4, APBB2, CAMK1D	19a: HIPK3^a^, MDM4, MAPK8, ADAMTS20, KLHL20 181a: HIPK3^a^, HSP90B1, UBE2B, TAF9B, DNAJC3
establishment of cell polarity	GO:0030010	RAB11FIP2, UST, RICTOR, WEE1, EPHB1, MARK1, KIF26B	19a: WEE1 181a: RAB11FIP2, MARK1
intrinsic apoptotic signaling pathway by p53 class mediator	GO:0072332	ZMAT1, ZMAT4, ZMAT3, PPP1R13B, ZNF385B, DDX5, ZNF346	19a: ZNF385B
negative regulation of extrinsic apoptotic signaling pathway	GO:2001237	PHIP, ZMYND11, NRP1, ITGA6, IGF1, PSME3, RPS6KB1, SGMS1, GCLM	19a: ZMYND11 181a: GCLM, RPS6KB1
histone ubiquitination	GO:0016574	SUZ12, UBE2A, HUWE1, UBE2B, PHC1	19a: UBE2A, SUZ12 181a: UBE2B
neuronal stem cell population maintenance	GO:0097150	NOTCH1, FOXO1, DLL1, FOXO3, CDH2, PROX1, MMP24	181a: PROX1
chromatin remodeling	GO:0006338	ATRX, TOP1, RSF1, HNF1A, GATA3, MORF4L2, INO80, CHD1, TP63, ARID1B, SMARCA2, RERE	19a: INO80^a^, SMARCA2, ATRX 181a: INO80^a^
positive regulation of cell-substrate adhesion	GO:0010811	PPM1F, SMOC2, CCDC80, JAK2, NID1, EDIL3, PRKCE, ABI3BP	19a: SMOC2 181a: ABI3BP
histone H3-K9 trimethylation	GO:0036124	BEND3, ARID4A, ARID4B	
histone H2A ubiquitination	GO:0033522	UBE2A, UBR2, UBE2B	19a: UBE2A 181a: UBE2B
positive regulation of cell proliferation	GO:0008284	KMT2D, CNBP, CACUL1, ESM1, IL34, CNOT7, CNOT6, TGFB2, PTK2, S1PR1, KRAS, CNOT6L, RARB, LOC488215, INSR, ACER3, UBE2A, PDCD10, KLB, SLC25A5, MECP2, ROGDI, IGF1, DLL1, TET1, SUZ12, ADM, HIPK1, HDAC1, VEGFA, HBEGF, MAB21L1, CARM1, EIF5A2	18a: KLB 19a: S1PR1^a^, CNOT7, CACUL1, NOT6, UBE2A, ROGDI, HIPK1, SUZ12 181a: S1PR1^a^, ESM1, CARM1, ADM
negative regulation of cell migration	GO:0030336	PTPRJ, RAP2A, ADARB1, RAP2C, OSBPL8, ABHD2, KANK1, THY1, SFRP1, ROBO1, RRAS, TP53INP1, SRGAP2	19a: RAP2C 181a: KANK1, OSBPL8, ADARB1
histone H3-K4 trimethylation	GO:0080182	TET3, BEND3, ARID4A, KMT2A, CTR9	181a: KMT2A
stem cell population maintenance	GO:0019827	PHF19, EIF4E, NIPBL, MED28, MTF2, EOMES, KLF4, DDX6, CTR9	18a: PHF19 19a: DDX6
cell migration	GO:0016477	CCDC88A, AVL9, CDH2, VAV2, EPHB3, SDC4, TGFB2, SDC1, SORBS2, GSK3B, ARF4, CDC42BPA, JAK2, LIMD1, LAMC1, CSK, KCTD13, NFATC2, USP33	18a: SDC4, SORBS2 19a: USP33^a^, NFATC2, CCDC88A, SDC1, EPHB3 181a: USP33^a^, AVL9, CDC42BPA

Gene symbols with superscripted letter (a) in last column denote gene targets associated with more than one miRNA

## Discussion

To the authors’ knowledge, this is the first study reporting secretion of exosome-like extracellular vesicles by canine mammary epithelial cells in vitro. Similar to previous reports of canine exosomes found in urine and serum/plasma, the vesicles were irregularly rounded, occasionally “cup-shaped,” and immunopositive for the transmembrane tetraspanin protein CD9, known to regulate the progression of cancer [37–39]. These findings strongly support these vesicles being exosomes, the small subcellular particles 50–200 nm in diameter that are actively shed from multivesicular bodies of parent cells. Exosomes contain proteins, peptides, mRNA, and microRNA, have been shown to be taken up by distinct cells through endocytosis, and they may play a role in distant cell-to-cell communication, especially in the context of neoplasia [37, 38].

As expected, the cell-free conditioned media that contained these exosomes was highly enriched in hundreds of distinct microRNAs. The microRNA profile of normal and malignant canine mammary exosomes was distinct, and yielded a number of significantly up-regulated and down-regulated miRs that may represent putative biomarkers of mammary neoplasia. These findings largely corroborate previous studies on miRNA in canine mammary neoplasia. Several studies of miRNA expression in canine mammary tumor tissue and CMT cells versus normal mammary tissue found significantly increased miR-29b, which was also significantly upregulated in the CMT exosomal RNA in the present study (along with the closely related miR-29c) [24, 25]. One of those studies also found miR-181b was significantly upregulated in the tubulopapillary carcinoma subtype [24].

Interestingly, our results differ substantially from those of von Deetzen et al. (2014), although it should be pointed out that in that study, the authors used miR-181b and miR-155 as housekeeping controls for qRT-PCR normalization, and both of those miRs appear to be upregulated in our data and previous studies of CMT [24, 26, 40]. Our results also diverge from Bulkowska et al. (2017), where several relevant up-regulated miRNA in the present study (such as miR-19a, miR-29b/c, and miR-181a) were shown to be down-regulated in malignant mammary carcinomas with metastasis [27]. This could be explained by the dramatic changes in tumor cell phenotype and gene expression in metastatic lesions compared to their matched primary tumor, such as occurs in the epithelial-to-mesenchymal transition (EMT) that has been documented in metastatic canine mammary carcinoma [41].

miR-126 was previously identified as a circulating biomarker of multiple canine cancers including mammary carcinoma, and it is up-regulated in our CMT exosomal RNA [28]. This would fit with the hypothesis that canine mammary carcinoma cells secrete exosomes containing miR-126 (among other miRNA) into circulation. However, the other putative biomarker in that study, miR-214, was strongly down-regulated in our CMT exosomal RNA. One possibility for this mismatch could include secretion of miR-214 by other cells than mammary carcinoma cells (i.e. cells of the immune system, stroma, or other organs). Another possibility is a mismatch between tumor cell, exosomal, and circulating microRNA profiles. Supporting this hypothesis, previous cell culture work in our lab has shown a complex relationship between exosomal miRNA profiles and the miRNA profile of the parent cell lines. Several miRs, including miR-18a, miR-19a, miR-29c, miR-181a, miR-215, miR-345, miR-371, and miR-1841, are up-regulated in both CMT parent cells and their exosomes [9, 40]. However, miR-31 and miR-34c had mixed expression patterns in the parent cells despite being uniformly upregulated in exosomes, and miR-495 was strongly down-regulated in all CMT parent cells while being up-regulated in the exosomal RNA population [9, 40]. This preliminary finding may suggest there is an active selection or enrichment process of particular miRNA within exosomes, or a negative feedback loop with the targets they regulate.

Many of these exosomal miRNAs were predicted to target dozens or hundreds of individual gene targets. Of particular note, miR-18a, miR-18b and miR-22 were highly up-regulated in the CMT exosomal RNA group and predicted to have an extremely high likelihood of targeting to 3’ UTR of the estrogen receptor ESR1α mRNA (miRDB score of 99 for miR-18a and miR-18b, and 87 for miR-22). A number of other additional miRNA, including miR-181a, miR-181b, miR-181c, miR-181d, miR-19a, miR-19b, miR-148b, miR-203, miR-323, miR-874, and miR-486 were also predicted to negatively regulate ESR1α, although with a lower probability than the > 80 score threshold (Additional file 4: Table S4). Although targets with score greater than 80 have a greater likelihood of being true targets, some targets with scores below 80 may also be real. While it has long been known that human and canine mammary neoplasms lose ESR1α expression along with increasing grade and stage, this finding may indicate that miRNA such as miR-18a contribute to this loss of hormone receptor activity [12, 15]. If this is verified in vivo in clinical patients, it may suggest miR-18a and others represent a non-invasive marker of CMT hormone status and phenotype, and could provide one potential mechanism for the loss of ESR1α with increasing CMT grade.

For gene ontology and functional enrichment analysis, the DAVID resource provides both *p*-values and Benjamini corrected *p*-values to aid investigators in the analysis process. The higher stringency of Benjamini corrected *p*-values dramatically reduces the number of significant results. On the one hand, this is a valuable way to reduce false positives from the analysis. On the other hand, a bioinformatics analysis aimed at providing clues as to the biological roles of miRNAs exhibiting altered expression profiles in normal mammary samples versus mammary tumor samples benefits greatly from broader inclusion criteria. Therefore, both the *p*-value and Benjamini corrected *p*-value were reported, and inclusion criteria for enriched gene ontology terms was set with a threshold *p*-value less than or equal to 0.06. This approach parallels the method described in Irizarry et al. (2016) and allows for retrieval of relevant functional annotation occurring at (or near) the boundary of *p*-value significance [36].

This set of enriched biological processes is particularly interesting in the context of mammary tumorigenesis and progression. The cell cycle-associated processes clearly relate to accelerated proliferation that can contribute to malignant transformation. For example, the transition between G1/S is a critically regulated check point in the cell cycle [42]. Aberrations in the control of G1/S transitions can contribute to aberrant cell division and undesirable cell proliferation [42]. Similarly, positive and negative regulation of apoptosis impacts which cells survive in the cellular population. Altered expression of pro-apoptotic and/or anti-apoptotic gene products may adversely contribute to increased susceptibility to tumorigenesis in mammary tissues.

Equally important are the biological processes associated with cellular differentiation, adhesion and stem cell maintenance. Molecular factors underlying cellular differentiation may contribute to altered genetic programs within the cell. Abnormal expression of these factors may alter cellular programs leading to dysregulation of the cell cycle. Likewise, altered levels of genes implicated in cell adhesion may contribute to metastatic phenotypes that shift the clinical status of tumors from benign to malignant.

Finally, biological processes regulating chromatin have tremendous potential to dramatically alter the long-term genetic programs associated with cells. Modification of histones through methylation, acetylation, deacetylation, and ubiquitination directly modulate which chromatin regions are accessible for gene expression [43]. Silenced regions encoding tumor suppressors may shift cells towards a more oncogenic potential [43].

This study has a number of important limitations. First, the number of biological replicates was relatively small, which was a function of both the high cost of the RNAseq methodology, as well as the difficulty harvesting and maintaining normal canine mammary epithelial cells in culture. However, the RNAseq dataset identified hundreds of miRs, many of which were significantly different, and many of these miRs match other studies in the human and veterinary literature. Another limitation is that there is currently no consensus as to the optimal way to normalize exosomal microRNA qRT-PCR data. This was dealt with through a commonly used approach of relative expression based on ΔΔ-Cq normalization to pooled endogenous (miR-16) and exogenous (cel-miR-39) controls, which yielded very similar fold-change between RNAseq and qRT-PCR (Fig. 3d). Furthermore, the use of specific stem-loop primers and sequence-specific probes, rather than non-specific intercalating dye methods (i.e. SYBR green), increased the specificity and robustness of this data.

## Conclusions

These data suggest that as in women with BC, CMT cells shed exosomes enriched in differentially expressed miRNA, especially miR-18a, miR-19a, and miR-181a. Preliminary in silico evidence suggests these miRNAs may modulate biological processes associated with, or contributing to, the balance between normal and neoplastic states. A miRNA population predicted to regulate so many aspects of cellular proliferation and hormone activity suggests that these miRs are not just inert cellular by-products, but may actually play an active part in neoplastic transformation and/or progression, and evidence that they are actively selected for secretion. Furthermore, the identification of these miRs in secreted exosomes raises the possibility that they may be shed into biofluids such as blood, urine, and breast milk, allowing their use as minimally-invasive biomarkers with mechanistic and prognostic relevance, and the similarity between canine and human breast cancer exosomal miRNA profiles may have significance for translational research, and future studies need to experimentally validate that these miRNAs regulate the predicted targets (such as miR-18a and ESR1α).

## Additional files

Additional file 1: **Table S1.** This table contains normalized RNAseq expression data for all miRNA in this study in Reads per Million (RPM). (XLSX 76 kb)

Additional file 2: **Table S2. **This table contains all genes predicted to be targeted and probability of binding their 3’ UTR for the set of 16 miRNA in Fig. 4. (XLSX 109 kb)

Additional file 3: **Table S3. **This provides a list of all Gene Ontology (GO) terms enriched in our set of 16 miRNA (Fig. 4). (XLSX 31 kb)

Additional file 4: **Table S4. **This table provides all miRNA in our study predicted to target the Estrogen Receptor ESR1, ranked by binding probability. (XLSX 10 kb)
